# Childhood sexual abuse is associated with higher total ghrelin serum levels in adulthood: results from a large, population-based study

**DOI:** 10.1038/s41398-023-02517-z

**Published:** 2023-06-22

**Authors:** Dirk Alexander Wittekind, Jürgen Kratzsch, Roland Mergl, Kerstin Wirkner, Ronny Baber, Christian Sander, A. Veronica Witte, Arno Villringer, Michael Kluge

**Affiliations:** 1 Institute of Laboratory Medicine, Clinical Chemistry and Molecular Diagnostics, University of Leipzig, Leipzig, Germany; 2 Institute of Psychology, University of the Bundeswehr Munich, Neubiberg, Germany; 3 Leipzig Research Center for Civilization Diseases (LIFE), University of Leipzig, Leipzig, Germany; 4 Institute for Medical Informatics, Statistics and Epidemiology, Leipzig University, Leipzig, Germany; 5 Department of Psychiatry and Psychotherapy, University of Leipzig, Leipzig, Germany; 6 Clinic of Cognitive Neurology, University of Leipzig, and Department of Neurology, Max Planck Institute for Cognitive and Brain Sciences, Leipzig, Germany; 7 Department of Psychiatry, Psychotherapy and Psychosomatics, Rudolf-Virchow-Hospital, Glauchau, Germany

**Keywords:** Depression, Biomarkers

## Abstract

Ghrelin is an orexigenic peptide hormone synthesized in times of stress and hunger and alterations of the ghrelin system following acute stressors could be repeatedly shown in humans. However, little data exists on long-term effects of trauma on the ghrelin system. We aimed to investigate the influence of childhood trauma on total ghrelin serum levels in a large, population-based study. Total serum ghrelin was measured in 1666 participants of a population-based cross-sectional study (‘LIFE study’). The Childhood Trauma Screener (CTS) was used for the assessment of childhood trauma in the final sample (*n* = 1086; mean age: 57.10 ± 16.23 years; 632 males, 454 females). Multiple linear regression analyses and generalized linear models were chosen to examine the association between childhood trauma and total serum ghrelin concentrations. Childhood sexual abuse went along with significantly higher ghrelin serum levels in the total sample (*β* = 0.114, *t* = 3.958; *p* = 0.00008) and in women (*β* = 0.142, *t* = 3.115; *p* = 0.002), but not in men (*β* = 0.055; *t* = 1.388; *p* = 0.166). Women with severe emotional neglect in the childhood had higher ghrelin levels than those without (odds ratio = 1.204; *p* = 0.018). For the CTS Sum Score and other CTS sub-scale scores, no significant association with ghrelin serum levels was found. Our study is the first to show associations between childhood sexual trauma and total ghrelin levels in adults in a large, community-based sample. Our results should initiate further research of the role of ghrelin in human stress response in prospective study designs.

## Introduction

Ghrelin is a 28-amino-acid peptide hormone that is predominantly synthesized in the stomach mucosa [1]. It was first discovered in 1999 as a strong stimulant of Growth-Hormone (GH) release [1] and binds to its almost ubiquitously expressed [2] receptor, the Growth Hormone Secretagogue Receptor (GHRS-1a) after a post-translational acylation by the enzyme Ghrelin O-Acyl Transferase (GOAT) [3, 4]. Ghrelin is secreted in a pulsatile manner [5] and secretion increases in times of stress and hunger [6]. It increases food intake especially of high palatable foods and induces weight gain [7]. Ghrelin has been repeatedly suggested to act as a survival hormone [8, 9], inducing both metabolic and behavioral adaptions to stress and hunger. It decreases energy expenditure [10] while promoting energy intake [11], thus acting towards energy homeostasis. Its involvement in stress response on a behavioral level is complex. While most animals studies show increased levels of serum ghrelin in acute [12–14] and chronic [15, 16] stress, ghrelin’s effects on behavior, i.e. if it acts depressiogenic/anxiogenic or antidepressant/anxiolytic seem to depend on a variety of factors, the most important of which seem to be acute vs. chronic exposure (for more information see [17]) and feeding state at the time of the stressor [18]. In humans, data is naturally more scarce, but acute stress exposure was mainly associated with increased serum levels [13, 19–21]. Interestingly, in one study in traumatized adolescents, ghrelin levels remained elevated even after the stressor had passed [14, 22]. This suggests that the ghrelin system shows stress-induced alterations considerable time after the stressor occurred. However, to date, it is widely unclear, how long and to what extent these changes persist. One recently published initial study in Anorexia nervosa (AN) patients found a positive association between childhood traumatization and ghrelin levels in their adult AN patients, providing a first indication that increased ghrelin levels can persist into adulthood [23]. Another study, investigating ghrelin’s effect on smoking and relapse in adult smokers, also found a positive association between childhood adverse events and ghrelin serum levels [24]. A very recent publication found a negative association of total ghrelin levels with childhood stress [25]. Childhood adverse events and trauma are known to have long lasting and often severe effects on mental and physical health well into adulthood [26]. Given the high prevalence of childhood trauma in the population [27], better understanding the underlying mechanisms which perpetrate health consequences into adulthood is important for developing new treatment approaches. Also, it has yet to be investigated whether the effects seen by Rossi et al. and Al’Absi et al. occur also in subjects who never developed a psychiatric illness despite experiencing trauma; i.e. whether the ghrelin system is also involved in “healthy” subjects or whether differences can only be seen in disease. Thus, we aimed to investigate the association between traumatic childhood experiences with total ghrelin serum levels in a large, population-based subject sample. Based on the results from previous research, we hypothesized that ghrelin levels would be positively associated with traumatic childhood experiences. Furthermore, we hypothesized that ghrelin levels would be positively associated with the intensity of traumatic childhood experiences. Explorative analyses addressed the question whether there was a significant association between the presence or intensity of specific childhood traumatic experiences and ghrelin serum levels.

## Methods

### Study design and subjects

Data for the present analysis come from the LIFE-Adult-Study (Leipzig Research Center for Civilization Diseases), a large population-based cohort study comprising 10,000 adults (mainly within the age range 40–79 years), who were randomly recruited in Leipzig, a large city in Germany [28]. All participants gave written informed consent to participate in the study. The study was conducted according to the Declaration of Helsinki and approved by the ethics committee of the University of Leipzig (registration-number: 263-2009-14122009). Blood samples were collected after an overnight fast (at least 8 h of fasting) including abstinence from smoking, between 07:30 and 10:30 h. The blood samples had been immediately processed by the team of the LIFE pre-analytical laboratory which is part of the Leipzig Medical Biobank and sent directly to the Institute of Laboratory Medicine, Clinical Chemistry, and Molecular Diagnostics (ILM) where direct analyses were carried out. Additional samples were frozen within 2 h after blood withdrawal in the vapor phase of liquid nitrogen at temperatures below −150 °C in Askion c-line HS200S (Askion, Gera, Germany) until usage for ghrelin measurements.

From the LIFE database, data sets were selected according to the following inclusion and exclusion criteria. Inclusion criteria were: valid measurements of total ghrelin; complete data regarding age, gender, alcohol consumption, nicotine consumption, BMI score, cortisol concentration, GAD-7 and CES-D sum scores; written informed consent and sufficient knowledge of the German language. Exclusion criteria (due to possible effects on ghrelin levels) were as follows: current treatment due to an autoimmune disease; treatment in the last year because of a diagnosis of cancer; current diseases of the gastrointestinal system; a history of stroke, multiple sclerosis or epilepsy. (Psycho-)pharmacological treatment was no principal exclusion criterion. Valid measurements of total ghrelin were available for 1666 LIFE participants. 580 individuals had to be excluded due to not meeting in- or exclusion criteria. The resulting final sample consisted of 1086 individuals (632 males and 454 females).

### Ghrelin measurements

All ghrelin measurements were done in serum by the use of a radioimmunoassay for total ghrelin (Mediagnost, Reutlingen Germany). Samples were not pre-treated with enzyme inhibitors or acidification. Due to this, only total ghrelin was measured, as it is much more stable than acyl-ghrelin. Sensitivity of the assay was 0.04 ng/mL, mean intra-assay coefficients of variation were 2.7–4.3%; interassay coefficients of variation were between 6.9 and 9.2% for the mean expected range of clinical data around 0.88 and 0.97 ng/mL.

### Cortisol measurements

Cortisol was measured in the same serum samples as ghrelin. Measurements were performed by the cobas® e601 fully automated system (Roche Diagnostics, Penzberg). Interassay coefficients of variation of 4.03% and 2.80% with cortisol levels between 65.6–69.2 nmol/L (*n* = 174) and 762–807 nmol/L (*n* = 174) were calculated after representative measurement of quality control sera over 6 months.

### Childhood Trauma Screener

Childhood trauma was assessed with the German version of the Childhood Trauma Screener (CTS), a 5-item self-rating screening tool [29]. Thus, childhood trauma has not been broadly assessed; this fact was due to the large number of questionnaires which had to be filled in within the LIFE-Adult Study. The questions assess emotional neglect (coded inversely), physical abuse, emotional abuse, sexual abuse, and physical neglect (coded inversely). The possible answers range from 1 (never) to 5 (very often) reflecting the intensity of the corresponding childhood trauma. For each item there is a cut-off that marks the presence or absence of the respective traumatization: For the sub-items physical and emotional abuse, the cut-offs were 3 (“occasionally”), for sexual abuse 2 (“rarely”), for emotional and physical neglect 4 (“frequently”). Furthermore, a sum-score can be calculated by addition of the five item scores. The correlations between the 5 items and the respective subscales of the more detailed Childhood Trauma Questionnaire (CTQ) range between *r* = 0.55 and *r* = 0.87 [30]. The CTS has a high internal consistency (Crohnach’s alpha 0.757) [29].

### Acquisition of data on tobacco and alcohol consumption

Tobacco consumption was assessed via self-administered questionnaire and interview. Subjects were grouped in three categories: active smoker, former smoker, and never-smoker. Active smokers were considered all those participants who had smoked regularly for at least 6 months consecutively in their lifetime and at least occasionally at the time of examination. Subjects who had smoked continuously for more than 6 months during their lifetime, but were not smoking at the time of assessment, were defined as former smokers [31].

Frequency and amount of consumption of alcoholic beverages (i.e. beer/wine/spirits) during the last 12 months were semiquantitatively assessed using a self-administered food frequency and alcohol questionnaire (FFQ). Possible answers for the frequency of alcohol consumption were “multiple times a day”, “daily”, “multiple times a week”, “once a week”, “two to three times a month”, “once a month or rarer”, or “almost never”. Also, the amount of beverage consumption was assessed by defined categories. From the amount and frequency of alcoholic beverage as well as the average alcohol content of different beverages, the average consumption of pure alcohol (g/day) was calculated [32].

### Assessment of BMI

The BMI is defined as the body weight divided by the square of the body height (kg/m2). Body weight was measured with an electronic scale (SECA 701, Seca GmbH & Co KG) with a precision of 0.01 kg, height using a stadiometer (SECA 240) to the nearest 0.1 cm by trained staff according to standardized protocols.

### Statistical analysis

In order to answer the question whether the findings of the study were due in part to other factors known to be associated with ghrelin serum concentrations like the BMI score Spearman-Brown correlations between ghrelin serum concentrations and covariates (age, BMI score, alcohol consumption (gram per day), cortisol levels, and GAD-7 and CES-D sum scores) were computed (due to non-normal distribution of the variables as assessed by the Kolmogorov–Smirnov test). In order to assess the association between ghrelin serum levels and binary variables (gender (male/female), smoking status (active smoking: yes versus no)) point-biserial correlation coefficients were chosen.

For primary analyses, generalized linear models with gamma distribution and log-link function were chosen to examine the association between presence of different childhood trauma types according to the CTS items 1–5 (reflecting emotional neglect, physical abuse, emotional abuse, sexual abuse, physical neglect: yes versus no) (as independent variables) and ghrelin serum concentrations (as dependent variable) because of a clearly skewed distribution of the ghrelin serum levels. The models were both unadjusted and adjusted for gender, age (continuous variable in years), BMI score, alcohol consumption (gram per day), smoking status, cortisol levels, GAD-7 and CES-D sum scores. The results were presented as odds ratios (ORs) with corresponding 95% confidence intervals (CI).

For subsequent subgroup analyses, we also applied generalized linear models with gamma distribution and log-link function to examine the gender-specific association between presence of different childhood trauma types and ghrelin serum concentrations (see above) with the effects of age, BMI score, alcohol consumption, smoking status, cortisol levels, GAD-7 and CES-D sum scores being controlled for. Corresponding unadjusted models were computed, too.

For secondary analyses, multivariate linear regression analyses were calculated for both the total sample and two subgroups (males and females). The independent variables were the CTS sum scores and the CTS items 1–5 (reflecting emotional neglect, physical abuse, emotional abuse, sexual abuse and physical neglect) and the dependent variable was the ghrelin concentration. The analyses were adjusted for age, gender, smoking status, alcohol consumption, BMI scores, cortisol levels, and GAD-7 sum scores [33] as well as the CES-D sum scores. Regression coefficients (β) with 95% confidence intervals (CI) were computed. For regression analyses in males and females, the same covariables were selected (except of gender).

Supplementary analyses were as follows:

Gender differences regarding ghrelin serum levels, CTS items and corresponding sum scores were assessed by using Mann–Whitney *U* tests in view of a non-normal distribution of the dependent variables (Kolmogorov–Smirnov test: *p* < 0.05).

Differences between individuals with versus without presence of different childhood trauma types according to the CTS items 1–5 (emotional neglect, physical abuse, emotional abuse, sexual abuse, physical neglect: yes versus no) regarding clinically relevant variables (CES-D sum scores, GAD-7 sum scores, BMI scores and alcohol consumption (in gram per day)) in the total sample were tested for statistical significance by using Mann–Whitney *U* tests. Moreover, two types of multiple regression analyses were chosen for supplementary statistical tests:

First, unadjusted multiple linear regression analyses were calculated for the total sample. The independent variables were the CTS items 1–5 (see above) and the dependent variable was the CES-D sum score. Thus, the association between the intensity of different childhood trauma types and the intensity of depression could be assessed. An analogous approach was selected for the GAD-7 sum score as dependent variable.

Second, unadjusted multiple regression analyses were computed for the total sample with the independent variables being the binary CTS items 1–5 (reflecting emotional neglect, physical abuse, emotional abuse, sexual abuse, and physical neglect: yes versus no; reference category: no) and the dependent variable being the CES-D sum score. Thus, the association between the presence of different childhood trauma types and the intensity of depression could be evaluated. An analogous approach was chosen for the GAD-7 sum score as dependent variable.

The frequency distribution of all 32 combinations of the binary CTS items 1–5 (representing emotional neglect, physical abuse, emotional abuse, sexual abuse, and physical neglect: yes versus no) was presented, too.

Moreover, the Bonferroni–Holm method was used to correct for multiple comparisons.

The SPSS version 26.0 was selected for the statistical analyses and the significance level *α* = 0.05 was chosen. All statistical tests were two-sided.

## Results

### Demographic and clinical characteristics of the sample

Demographic characteristics (age and gender distribution) as well as clinical data of the final sample (like severity of anxiety and depression) are summarized in Table 1.

**Table 1:** Description of the sample.

Variable	Total sample (*n* = 1086)	Males (*n* = 632; 58.20%)	Females (*n* = 454; 41.80%)	*p*-value
Ghrelin serum level, pg/ml, mean (SD)	904.98 (436.33)	805.53 (314.29)	1043.43 (534.24)	<0.001***^a^
Age, years, mean (SD)	57.10 (16.23)	57.33 (16.49)	56.79 (15.86)	0.204^a^
BMI, kg/m^**2**^, mean (SD)	27.00 (4.49)	27.27 (4.05)	26.63 (5.02)	<0.001***^a^
Alcohol consumption, g/day, mean (SD)	12.99 (17.79)	18.29 (20.31)	5.61 (9.47)	<0.001***^a^
Non-smokers, *n* (%)	937 (86.3%)	530 (83.9%)	407 (89.6%)	0.006**^b^
Cortisol concentration, nmol/l, mean (SD)	512.61 (195.95)	496.30 (145.11)	535.30 (248.49)	0.904^a^
GAD-7 sum score, mean (SD)	2.70 (2.70)	2.44 (2.60)	3.05 (2.80)	<0.001***^a^
CES-D sum score, mean (SD)	9.22 (6.05)	8.59 (5.27)	10.08 (6.91)	0.0013**^a^

Significant findings were marked in bold.

*BMI* body mass index; *CES-D* Center for Epidemiological Studies Depboression Scale [56, 57] *CI* confidence interval; *GAD-7* Generalized Anxiety Disorder 7-item Scale [58, 59]; *SD* standard deviation.

*p ≤ 0.05; **p ≤ 0.01; ***p ≤ 0.001.

^a^Due to non-normal distribution of the dependent variables (Kolmogorov–Smirnov-Test: *p* < 0.05), a Mann–Whitney *U* test was computed in order to calculate *p*-values regarding ghrelin serum concentrations (*Z* = −9.093), age (*Z* = −1.271), BMI scores (*Z* = −3.483), alcohol consumption (*Z* = −14.041), cortisol concentration (*Z* = −0.121), GAD-7 sum scores (*Z* = −3.797), and CES-D sum scores (*Z* = −3.214).

^b^A *χ*^2^ test for a two-by-two cross table (*χ*^2^ = 7.47; df = 1) was applied.

### Childhood trauma characteristics of the sample

In all, 345 of 1086 reported having experienced childhood trauma (31.77%). Of the total sample, 81 participants (7.46%) had conspicuous CTS scores reflecting emotional neglect. Corresponding numbers were 91 (8.38%) for physical abuse, 77 (7.09%) for emotional abuse, 60 (5.52%) for sexual abuse and 203 (18.69%) for physical neglect.

The frequency distribution of all 32 combinations of the binary CTS items 1–5 representing emotional neglect, physical abuse, emotional abuse, sexual abuse and physical neglect (yes versus no) was summarized in Table S3. The most frequent configuration (besides of absence of any childhood trauma (68.23%)) was physical neglect without further childhood traumas (12.98%), followed by physical abuse without further childhood traumas and sexual abuse without further childhood traumas (3.04% in both cases) The most frequent combination of two traumas was the combination of severe emotional neglect and physical neglect (1.66%), the most frequent triple combination that for severe emotional neglect, physical abuse, and emotional abuse (0.83%) and the most frequent quadruple combination that for severe emotional neglect, physical abuse, emotional abuse, and physical neglect (0.92%). All five childhood traumas were found in only four individuals (0.37%).

Detailed information on the mean intensity of different childhood trauma types and corresponding gender differences were presented in Table 2. Overall, the proportions of individuals with medium/severe emotional abuse and sexual abuse were significantly higher in females than in males (*p* ≤ 0.019).

**Table 2:** Childhood trauma characteristics of the sample.

Variables	Total sample (*n* = 1086)	Men (*n* = 632)	Women (*n* = 454)	*p-*value
CTS sum score (mean (SD))^a^	7.75 (2.66)	7.79 (2.47)	7.69 (2.91)	0.051^+b^
Emotional neglect (mean (SD))	4.18 (0.93)	4.13 (0.91)	4.25 (0.94)	0.004**^b^
Physical abuse - (mean (SD))	1.33 (0.75)	1.33 (0.74)	1.33 (0.77)	0.684^b^
Emotional abuse - (mean (SD))	1.27 (0.71)	1.22 (0.63)	1.33 (0.80)	0.047*^b^
Sexual abuse (mean (SD))	1.08 (0.38)	1.03 (0.23)	1.16 (0.52)	<0.001***^b^
Physical neglect (mean (SD))	3.75 (1.37)	3.67 (1.38)	3.87 (1.35)	0.009**^b^
Emotional neglect^c^ (*n*(%))	81 (7.5%)	47 (7.4%)	34 (7.5%)	0.974^d^
Physical abuse^c^ (*n*(%))	91 (8.4%)	52 (8.2%)	39 (8.6%)	0.832^d^
Emotional abuse^c^ (*n*(%))	77 (7.1%)	35 (5.5%)	42 (9.3%)	0.019*^d^
Sexual abuse^c^ (*n*(%))	60 (5.5%)	15 (2.4%)	45 (9.9%)	<0.001***^b^
Physical neglect^c^ (*n*(%))	203 (18.7%)	121 (19.1%)	82 (18.1%)	0.651^d^
Any childhood trauma^c^ (*n*(%))^c^	345 (31.8%)	192 (30.4%)	153 (33.7%)	0.246^d^

Significant findings were marked in bold.

*CTS* Childhood Trauma Screener [29], *n* sample sizes, *SD* standard deviation.

^+^*p* ≤ 0.10; *p ≤ 0.05; **p ≤ 0.01; ***p ≤ 0.001.

^a^ For computing the CTS sum score, inverse coding of the CTS items 1 and 5 was applied.

^b^ Due to a non-normal distribution of the dependent variables (Kolmogorov–Smirnov–Test: *p* < 0.05), a Mann–Whitney *U* test was computed in order to calculate *p* values regarding gender differences.

^c^ Presence of a childhood trauma was defined as follows: CTS item 1 (inverse coding) ≥4 or CTS item 2 ≥ 3 or CTS item 3 ≥ 3 or CTS item 4 ≥ 2 or CTS item 5 (inverse coding) ≥4.

^d^ A *χ*^2^ test for a two-by-two cross table was computed.

### Association between ghrelin serum levels and presence of childhood trauma

Ghrelin serum concentrations were found to be significantly associated with the following variables in the total sample: gender (*r* = 0.269; *p* < 0.001), age (*ρ* = −0.110; *p* < 0.001), the BMI score (*ρ* = −0.284; *p* < 0.001) and the GAD-7 sum score (*ρ* = 0.102; *p* < 0.001). There was a statistical trend regarding the positive association of ghrelin serum concentrations and the cortisol concentrations (*ρ* = 0.051; *p* = 0.09). The associations between ghrelin serum concentrations and active smoking (*r* = 0.019; *p* = 0.54), alcohol consumption (*ρ* = −0.020; *p* = 0.51) and the CES-D sum score (*ρ* = 0.004; *p* = 0.89) failed to be statistically significant in the total sample.

Overall, presence of at least one childhood trauma did not go along with significantly higher total ghrelin serum levels as compared to individuals without such experiences (unadjusted model: OR = 1.014; *p* = 0.609; adjusted model: OR = 0.999; *p* = 0.964; see also Table 3).

**Table 3:** Association between presence of different childhood trauma types and ghrelin serum levels in the total sample (*n* = 1086).

Trauma type	*n*	Odds ratio [95% CI] (*p-*value)	
		Model 1	Model 2
Any childhood trauma	345	1.014 [0.961–1.070] (*p* = 0.609)	0.999 [0.949–1.051] (*p* = 0.964)
Severe emotional neglect	81	**1.100 [1.000–1.210] (*p*** = 0.049*)	1.070 [0.979–1.169] (*p* = 0.138)
Physical abuse	91	0.994 [0.908–1.088] (*p* = 0.895)	0.978 [0.898–1.064] (*p* = 0.599)
Emotional abuse	77	1.017 [0.923–1.122] (*p* = 0.732)	0.977 [0.891–1.072] (*p* = 0.620)
Sexual abuse	60	**1.217 [1.091–1.357] (*p*** = 0.0004***)	1.098 [0-990-1.217] (*p* = 0.076^+^)
Physical neglect	203	1.000 [0.938–1.066] (*p* = 0.999)	1.015 [0.954–1.080] (*p* = 0.639)

Significant findings were marked in bold. All statistical models were generalized linear models with gamma distribution and log-link function for the examination of the association of presence of a childhood trauma and different types of childhood trauma (emotional neglect, physical abuse, emotional abuse, sexual abuse, physical neglect; reference category: no) and ghrelin serum levels (as dependent variable). Model 1 was unadjusted. Model 2 was adjusted for age (years), gender (reference category: male), body mass index (BMI) scores, alcohol consumption (gram/day), smoking status (reference category: non-smokers), cortisol levels, GAD-7 sum scores, and CES-D sum scores.

*CI* confidence interval, *n* sample sizes.

^+^*p* ≤ 0.10; *p ≤ 0.05; **p ≤ 0.01; ***p ≤ 0.001.

Presence of a childhood trauma was defined as follows: CTS item 1 (inverse coding) ≥4 or CTS item 2 ≥ 3 or CTS item 3 ≥ 3 or CTS item 4 ≥ 2 or CTS item 5 (inverse coding) ≥4. For all other cases, absence of a childhood trauma was assumed.

For severe emotional neglect, a significantly positive association with ghrelin serum levels was found in an unadjusted model (OR = 1.100; *p* = 0.049); however, this association was not significant in a generalized linear model, corrected for several covariables (age, gender, BMI scores, alcohol consumption, smoking status, cortisol levels, GAD-7 and CES-D sum scores) (OR = 1.070; *p* = 0.138). Physical abuse, emotional abuse and physical neglect were neither significantly associated with ghrelin serum levels in unadjusted models nor adjusted models (see Table 3). Instead, individuals with sexual abuse had significantly higher total ghrelin serum levels than individuals without sexual abuse in an unadjusted model (OR = 1.217; *p* = 0.0004); a corresponding statistical tendency was found in the adjusted model (OR = 1.098; *p* = 0.076).

Gender-specific subgroup analyses revealed that presence of childhood trauma and ghrelin serum levels were not significantly associated in males (see Table 4). Regarding females, severe emotional neglect went along with significantly higher ghrelin serum levels at the 5% level (unadjusted model: OR = 1.232; *p* = 0.009; adjusted model: OR = 1.204; *p* = 0.018). The latter OR did not significantly differ from the corresponding OR in males (0.965) as revealed by the overlapping 95% confidence intervals (males: 0.868–1.072; females: 1.032–1.405).

**Table 4:** Association between presence of different childhood trauma types and ghrelin serum levels in males (*n* = 632) and females (*n* = 454).

Trauma type	*n*	Odds ratio [95% CI] (*p* value)	
		Model 1	Model 2
Males (*n* = 632)			
Any childhood trauma	192	0.976 [0.918–1.037] (*p* = 0.427)	0.974 [0.915–1.037] (*p* = 0.405)
Severe emotional neglect	47	0.978 [0.879–1.089] (*p* = 0.689)	0.965 [0.868–1.072] (*p* = 0.507)
Physical abuse	52	0.941 [0.849–1.042] (*p* = 0.241)	0.931 [0.843–1.029] (*p* = 0.164)
Emotional abuse	35	0.938 [0.830–1.060] (*p* = 0.307)	0.930 [0.825–1.049] (*p* = 0.240)
Sexual abuse	15	1.179 [0.981–1.417] (*p* = 0.079^+^)	1.138 [0.949–1.364] (*p* = 0.162)
Physical neglect	121	0.992 [0.924–1.065] (*p* = 0.823)	1.007 [0.936–1.083] (*p* = 0.859)
Females (*n* = 454)			
Any childhood trauma	153	1.034 [0.947–1.130] (*p* = 0.458)	1.019 [0.935–1.112] (*p* = 0.665)
Severe emotional neglect	34	**1.232 [1.052–1.442] (*p*** = 0.009**)	**1.204 [1.032–1.405] (*p*** = 0.018*)
Physical abuse	39	1.043 [0.899–1.211] (*p* = 0.578)	1.047 [0.904–1.212] (*p* = 0.538)
Emotional abuse	42	1.011 [0.875–1.168] (*p* = 0.883)	1.013 [0.876–1.171] (*p* = 0.861)
Sexual abuse	45	1.099 [0.956–1.264] (*p* = 0.184)	1.076 [0.941–1.231] (*p* = 0.285)
Physical neglect	82	1.019 [0.914–1.136] (*p* = 0.737)	1.012 [0.907–1.128] (*p* = 0.836)

Significant findings were marked in bold. All statistical models were generalized linear models with gamma distribution and log-link function for the examination of the association of presence of a childhood trauma and different types of childhood trauma (emotional neglect, physical abuse, emotional abuse, sexual abuse, physical neglect; reference category: no) and ghrelin serum levels (as dependent variable). Model 1 was unadjusted. Model 2 was adjusted for age (years), body mass index (BMI) scores, alcohol consumption (gram/day), smoking status (reference category: non-smokers), cortisol levels, GAD-7 sum scores, and CES-D sum scores.

*CI* confidence interval, *n* sample sizes.

^+^*p* ≤ 0.10; *p ≤ 0.05; **p ≤ 0.01; ***p ≤ 0.001.

Presence of a childhood trauma was defined as follows: CTS item 1 (inverse coding) ≥4 or CTS item 2 ≥ 3 or CTS item 3 ≥ 3 or CTS item 4 ≥ 2 or CTS item 5 (inverse coding) ≥4. For all other cases, absence of a childhood trauma was assumed.

Regarding the presence of sexual abuse, there was a positive association with ghrelin serum levels in both males and females but failed to be significant (*p* ≥ 0.08). When comparing medium to severe (CTS item sexual abuse of 3–5) with no or little childhood sexual trauma (CTS item sexual abuse of 1–2), i.e. when the cut-offs were set differently, results were significant at the 5% level (adjusted model: OR = 1.195; *p* = 0.02).

Physical abuse, emotional abuse and physical neglect in females were neither significantly associated with ghrelin serum levels in unadjusted models nor adjusted models (see Table 4).

### Association between ghrelin serum levels and the intensity of childhood abuse

In the total sample (*n* = 1086), ghrelin serum levels did not show a significant association with the intensity of emotional neglect, physical abuse, emotional abuse and physical neglect as measured by the CTS items 1–3 and 5 when adjusted for age, gender, BMI, alcohol consumption, smoking status, cortisol concentrations, GAD-7 sum scores, and CES-D sum scores (see Table 5, upper part). The same was true for the subgroups of males (see Table 5, middle part) and females (see Table 5, lower part). Whereas there was no significant association between the presence of childhood sexual abuse and ghrelin serum concentrations in adjusted generalized linear models, there was a significantly positive association between the intensity of sexual abuse (as reflected by the CTS item 4) and ghrelin serum concentrations in the total sample (ß = 130.01 (95% CI: 65.55; 194.47), standardized *β* = 0.114, *t* = 3.958; *p* = 0.00008; adjusted for the above mentioned covariables) (see Table 5, upper part) and in the female subgroup (ß = 145.35 (95% CI: 53.64; 237.06), standardized *β* = 0.142, *t* = 3.115; *p* = 0.002; adjusted for the above mentioned covariables without gender; see Table 5, lower part) whereas the corresponding positive association in the male subgroup (ß = 76.08 (95% CI: −31.56; 183.72), standardized *β* = 0.055; *t* = 1.388; see Table 5, middle part) failed to be statistically significant (*p* = 0.166).

**Table 5:** Results of multiple linear regression analyses regarding the association between ghrelin serum levels and CTS scores reflecting the intensity of childhood abuse.

Variables	Sample	Regression coefficient *β* (95% CI)	stand. ß	*t*	*p*-value
CTS sum score^**a**^	Total^b^	2.652 (−7.024; 12.33)	0.016	0.538	0.591
	Males^c^	−3.110 (−13.45; 7.23)	−0.024	−0.591	0.555
	Females^c^	7.674 (−9.830; 25.178)	0.042	0.862	0.389
Severe emotional neglect	Total^b^	4.513 (−22.72; 31.75)	0.010	0.325	0.745
	Males^c^	−12.47 (−39.68; 14.74)	−0.036	−0.900	0.369
	Females^c^	24.016 (−29.408; 77.440)	0.042	0.883	0.377
Physical abuse	Total^b^	−9.490 (−42.51; 23.53)	−0.016	−0.564	0.573
	Males^c^	−20.313 (−53.422; 12.797)	−0.048	−1.205	0.229
	Females^c^	7.551 (−57.211; 72.312)	0.011	0.229	0.819
Emotional abuse	Total^b^	3.950 (−31.45; 39.35)	0.006	0.219	0.827
	Males^c^	−4.260 (−42.939; 34.419)	−0.009	−0.216	0.829
	Females^c^	9.867 (−53.043; 72.778)	0.015	0.308	0.758
Sexual abuse	Total^b^	130.011 (65.55; 194.47)	0.114	3.958	<0.001**
	Males^c^	76.078 (−31.559; 183.716)	0.055	1.388	0.166
	Females^c^	145.348 (53.636; 237.060)	0.142	3.115	0.002**
Physical neglect	Total^b^	−1.143 (−20.47; 18.18)	−0.004	−0.116	0.908
	Males^c^	0.911 (−18.127; 19.950)	0.004	0.094	0.925
	Females^c^	−6.779 (−45.16; 31.60)	−0.017	−0.347	0.729

Significant findings were marked in bold.

*β* Regression coefficient, *BMI* body mass index, *CES-D* Center for Epidemiological Studies Depression Scale [56, 57], *CI* confidence interval, *CTS* Childhood Trauma Screener [29], *GAD-7* Generalized Anxiety Disorder 7-item Scale [58, 59], *N/n* sample sizes.

^+^*p* ≤ 0.10; *p ≤ 0.05; **p ≤ 0.01; ***p ≤ 0.001.

^a^For computing the CTS sum score, inverse coding of the CTS items 1 and 5 was applied.

^b^All multivariate linear regression models have been adjusted for age, gender, alcohol consumption, smoking status (1 = active smoking; 0 = non-smoking), BMI scores, cortisol concentrations, GAD-7 sum scores and CES-D sum scores.

^c^All multivariate linear regression models have been adjusted for age, alcohol consumption, smoking status (1 = active smoking; 0 = non-smoking), BMI scores, cortisol concentrations, GAD-7 sum scores and CES-D sum scores.

We analyzed to what extent distinct trauma types were associated with higher scores in depression (CES-D) and anxiety scores (GAD-7). When associating the presence of childhood abuse with outcomes in the CES-D and GAD-7, we found that for depressive symptomatology, emotional abuse (standardized *β* = 0.088; *t* = 2.454; *p* = 0.014), sexual abuse (standardized *β* = 0.085; *t* = 2.796; *p* = 0.005), and severe physical neglect (standardized *β* = 0.123; *t* = 4.036; *p* = 0.00006) were significantly and positively associated with CES-D sum scores. The two latter findings were also significant after alpha-adjustment. Similarly, in generalized anxiety the following categories were significantly and positively associated with GAD-7 sum scores: emotional abuse (standardized *β* = 0.077; *t* = 2.130; *p* = 0.033), sexual abuse (standardized *β* = 0.066; *t* = 2.157; *p* = 0.031), and severe physical neglect (standardized *β* = 0.083; *t* = 2.690; *p* = 0.007; see Table S2). The latter result was also significant at the alpha-adjusted significance level (*α* = 0.01).

When associating the intensity of different childhood trauma types with outcomes in the CES-D and GAD-7 it could be shown that emotional neglect (standardized *β* = 0.110; *t* = 3.221; *p* = 0.001), emotional abuse (standardized *β* = 0.099; *t* = 2.748; *p* = 0.006), sexual abuse (standardized *β* = 0.084; *t* = 2.765; *p* = 0.006) and physical neglect (standardized *β* =*β* = 0.092; *t* = 2.971; *p* = 0.003) were significantly and positively associated with CES-D sum scores. In contrast, only the intensity of emotional abuse (standardized *β* = 0.126; *t* = 3.442; *p* = 0.001) and sexual abuse (standardized *β* = 0.061; *t* = 1.994; *p* = 0.046) were significantly associated with higher GAD-7 sum scores. The latter finding was no longer significant after Bonferroni correction.

## Discussion

In this study we showed that childhood sexual abuse was positively associated with ghrelin serum levels in the total sample (standardized *β* = 0.114, *t* = 3.958; *p* = 0.00008) and in women (standardized *β* = 0.142, *t* = 3.115; *p* = 0.002), but not in men (standardized *β* = 0.055; *t* = 1.388; *p* = 0.166). Women who had experienced severe emotional neglect had higher ghrelin serum levels than those who did not (adjusted model: OR = 1.204; *p* = 0.018). For CTS Sum Score and the other sub-scores, no significant association or differences in serum levels were found.

The difference in total ghrelin serum levels between subjects with and without childhood sexual abuse showed a trend significance and we could show a positive association in a linear regression analysis. Thus, we decided to isolate subjects who had experienced medium to severe sexual abuse and compare them to subjects with no or little sexual abuse. Here, we found a statistically significant difference in the total group (adjusted model: OR = 1.195; *p* = 0.02), but not in men or women when analyzed separately. This is most likely a statistical effect due to relatively small sample sizes. This, together with the results from the regression analysis, suggests that the intensity of abuse has an influence on ghrelin serum levels.

We found considerable sex differences in our sample concerning both the regression analysis and the generalized linear models. Despite the large sample size, the percentage of subjects who had experienced sexual abuse and severe emotional neglect was quite low. Also, many more women were subject to abuse than men, so that women were overrepresented in the group with childhood abuse experience. Furthermore, in the regression analysis, there is considerable overlap between the 95%-confidence intervals of men (31.56–183.72) and women (53.64–237.06), which strongly suggests that the statistical difference is not a consequence of sex-specific differences in the ghrelin signal. Thus, we interpret the sex differences most likely as a statistical effect due mainly to the fact that group sizes were larger in women. However, sex-specific effects of ghrelin in stress regulation have been reported in animal studies, suggesting higher ghrelin sensitivity and stronger ghrelin effects in stress response in females [34]. Sex-specific differences in ghrelin signaling in feeding response has been shown in mice [35] and humans [36] and ghrelin-dependent growth hormone secretion was shown to be different in male and female mice [37]. Thus, an innate influence of sex on our results cannot be excluded, even if we found no traces of it in our statistical analysis. Further studies that take sex into account when analyzing stress response in humans are needed to investigate this question.

Our results are largely in line with the little evidence that exists on this subject. In a first study, Yousufzai and colleagues could show that children who had suffered traumatic experiences (*n* = 49) had higher acyl serum levels than the control group (*n* = 39), even years after the traumatic event happened [22]. Associated with higher ghrelin levels were increased scores in depressive and anxiety symptomatology and disturbed sleeping behavior [22]. In line with this, in our sample, subjects who suffered emotional abuse, sexual abuse or severe physical neglect had higher scores for depressive (as assessed in the CES-D) and anxiety (as assessed in the GAD-7) symptomatology (see supplementary material), underlining the prolonged negative effect that childhood abuse has on mental health. This finding is supported by recent literature [38, 39]. Comparability of the data published by Yousufzai et al with our sample is, however, reduced due to the fact that subjects were children, predominantly male, only acyl-ghrelin was assessed and blood samples were taken in the afternoon with no standardized meal/fasting period beforehand. From the same group and sample, another analysis showed that subjects with Posttraumatic Stress Disorder (PTSD) had higher acyl ghrelin serum levels than subjects who had experienced trauma but had not developed a PTSD and healthy controls [40]. In our sample, data on the presence of PTSD was only present in 15 subjects, of which only one subject fulfilled criteria of PTSD, so a statistical analysis could not be performed.

In another recent study, authors investigated the association between childhood trauma and total ghrelin serum levels in a sample of female Anorexia Nervosa (AN) patients and healthy controls [23]. Ghrelin was collected, as in our study, after a 12h- overnight fast in the morning. Interestingly, they found that, as in our sample, sexual abuse was (among emotional abuse in their sample) the strongest predictor for higher total ghrelin serum levels. Rossi et al found that physical neglect and total CTQ score were also positively associated with ghrelin serum levels, but effects were markedly lower than for emotional and sexual abuse [23]. This is of note, as it suggests, together with our data, that these types of traumas seem to be associated the strongest with ghrelin serum levels.

Al’Absi and colleagues found that high, but not low or medium, early life adversity [24] was associated with higher total ghrelin serum levels in non-smokers. This finding is relevant, as it backs up our observation that ghrelin serum levels were associated with the intensity of childhood abuse and effects were clearer in subjects with medium to severe childhood abuse [24]. Summing up, the research published so far investigates mainly specific subject samples (traumatized adolescents, AN-patients, smokers), but results are in line with ours from a broad, population-based approach.

Daniels et al investigated the association between early childhood stress and (ELS) and total ghrelin serum levels in young adults [25]. It is the first study to date that found a negative association between ghrelin levels and ELS. However, when the authors corrected for currents psychiatric condition, this association was no longer statistically significant. Results in our study were controlled for depression and anxiety scores as these are known to be associated with both ghrelin levels and childhood trauma [17, 38, 39]. Furthermore, they investigated much younger subjects (mean age 26.6 in control group, 27.8 years in group with ELS) and ghrelin serum levels were much lower than in our sample in general (~250 pg/ml vs ~800 pg/ml in our sample), suggesting differences in the total ghrelin assay that was used [25].

Ghrelin’s role in stress regulation has been subject of intensive animal research and the accumulating evidence paints a picture of a complex, non-linear involvement in stress response regulation, depending on a variety of factors, but mainly on whether ghrelin signaling is acute or chronic and whether animals were stressed or unstressed previous to ghrelin administration [13, 14]. Higher baseline ghrelin levels led to decreased fear memories in unstressed rodents [41] and to reduced aversive behavior following elevated ghrelin signaling, either via overexpression of GHRS-1a in the basolateral complex of the amygdala (BLA) [42] or via ghrelin administration in the amygdala [18]. Similarly, ghrelin administration in acute stress led to anxiolytic and antidepressant effects in animals [16, 43, 44]. However, in chronically stressed animals, ghrelin signaling seems to have the opposite effect [14, 41]. Interestingly, in chronic stress, GHRS-1a receptor expression was reduced in the BLA, the region whose activation led to decreased fear memory in unstressed rodents, suggesting a decreased ghrelin-sensitivity in chronic stress [41]. Other studies point in the same direction [45, 46]. This led Stone et al to hypothesize that ghrelin has a protective role in acute stress and a detrimental role in chronic stress [14].

Applied to our sample, elevated ghrelin levels years after stress events suggest a chronically increased ghrelin signal. In fact, as already discussed above, subjects who experienced severe emotional neglect, sexual abuse or emotional abuse all exhibited higher anxiety and depression scores. Given the cross-sectional design of our study, no causal conclusions can be drawn, but from what we know from animal studies and the human studies to date, an involvement of ghrelin seems at least plausible. Further, longitudinal studies are needed to clarify this.

The possible clinical relevance of ghrelin’s involvement in stress and mood regulation has been recently illustrated with ghrelin-based therapeutic approaches to psychiatric conditions. Ghrelin agonists were shown to have antidepressant effects in rodents [47] and for alcohol addiction, drug candidates have been used in humans with promising results [48]. Also, ghrelin measurement might be suitable to differentiate depression subtypes [49] and predict treatment response [50, 51]. Stone et al. [14] hypothesize that in future, characterization of ghrelin activity in humans suffering from PTSD and/or MDD might identify subjects that would profit from a ghrelin-based therapy.

In our analysis, not all categories of traumatic experiences were associated with increased ghrelin serum levels. We could show this for sexual abuse in the regression analysis and for severe emotional neglect in the generalized linear models for women. In multiple cohorts, one in the USA and one in Germany, combat exposure and sexual abuse was associated with significantly higher rates of PTSD than other trauma types [52, 53], suggesting more pronounced detrimental long-term effects. This in line with the finding of an association between ghrelin serum levels and PTSD in war-traumatized adolescents [40]. In a German sample, the chance of developing PTSD was twice as high in subjects experiencing sexual violence when compared to non-sexual violence [53]. A recent study investigating heavily traumatized women also found more pronounced negative effects after sexual violence as compared to non-sexual violence [54]. Thus, of the trauma categories in the screening tool used here, sexual abuse can be seen as the most severe trauma type. This might be one reason why ghrelin serum levels were predominantly associated with sexual abuse. This is line with other, similar studies: sexual abuse was also most strongly associated with ghrelin serum levels in another study and physical abuse was, as in our sample, not associated with ghrelin levels [23]. In summary, sexual abuse is most reliably associated both with negative psychological effects and endocrine changes, suggesting that it might be more destructive than other trauma types and be comparable to war- and combat exposure.

Childhood abuse is a heterogeneous and complex phenomenon and often, subjects did not just experience one type of traumatization in an isolated way. To be able to better interpret our results, we analyzed the frequency distribution of all possible combinations of the CTS items (Table S3). Of the subjects who experienced trauma, 33% experienced two or more types of trauma. This illustrates that the co-occurrence of different types of abuse is common. A slightly higher fraction was observed in the 60 subjects experiencing sexual trauma, of whom 27 also experienced other forms of abuse (45%). Thus, in this cross-sectional design, it cannot be fully excluded that the effects seen for sexual trauma might be due to the fact that subjects who experienced sexual abuse might represent a sub-group which is more heavily traumatized in general.

The rate of childhood abuse in total (31.77%) and of the subcategories are in line with other studies of comparable populations. For example, in another population-based cohort from Germany, in the city of Greifswald [30, 55], 4.3% of subjects reported childhood sexual abuse (vs. 5.52% in our sample). This suggests that our sample was in fact representative and we did not investigate a selected or biased group of subjects.

The main strengths of this investigation were the large sample size and the highly standardized data acquisition. Furthermore, in contrast to other studies, we strictly controlled our statistical analysis for factors known to influence the ghrelin serum levels, most importantly in this context anxiety scores [17], but also alcohol consumption [32] and smoking [31].

The cross-sectional design is the main limitations of this study. As a consequence, all hormones were only assessed at one time point. Also, only total and not acylated ghrelin was measured and blood samples were not pre-treated with protease inhibitors or acidification. Results were not controlled for current medication. Depression and anxiety measures were only assessed by self-rating instruments, childhood traumatic experiences were only assessed by a screening instrument and recent stressors were not assessed in our analysis.

In conclusion, we could show a positive association between childhood traumatic experiences and total serum ghrelin levels in adulthood for the first time in a large, population-based subject sample. Although causative conclusions cannot be drawn, our data suggests that the ghrelin system may be altered years and decades after traumatic experiences, lending further support for the relevance of the ghrelin system in stress response and – processing.

## Supplementary information

Supplementary Tables

## Data Availability

The data that support the findings of this study are available from the Leipzig Research Center for Civilisation Diseases (LIFE) but restrictions apply to the availability of these data, which were used under license (project number: PV_0358_Kluge) for the current study, and so are not publicly available. Data are however available from the authors upon reasonable request and with permission of LIFE.
