# Supplementary material for: Childhood sexual abuse is associated with higher total ghrelin serum levels in adulthood: results from a large, population-based study
Source: Transl Psychiatry. 2023 Jun 22;13:219. doi: 10.1038/s41398-023-02517-z (PMC10287627; doi:10.1038/s41398-023-02517-z)
Supplement: Supplementary file 1 — Supplementary Tables [file 41398_2023_2517_MOESM1_ESM.pdf]

Table S1: Differences between individuals with versus without presence of different childhood trauma types regarding clinically relevant variables in the total sample (N=1086)

Category	CES-D sum score (means (SD))	GAD-7 sum score (means (SD))	BMI score (means (SD))	Alcohol consumption (gram per day) (means (SD))
**CTS item 1 (inverse coding): Severe emotional neglect**				
**no (n=1005)**	9.07 (5.97)	2.64 (2.66)	26.98 (4.49)	12.82 (17.45)
**yes (n=81)**	11.06 (6.73)	3.35 (3.13)	27.18 (4.45)	15.04 (21.57)
**p value**	**0.005****	**0.042***	0.695	0.389
**CTS item 2: Physical abuse**				
**no (n=995)**	9.06 (5.78)	2.63 (2.62)	26.99 (4.53)	13.04 (17.82)
**yes (n=91)**	10.92 (8.33)	3.42 (3.40)	27.13 (4.00)	12.46 (17.56)
**p value**	0.079^{+}	**0.028***	0.541	0.306
**CTS item 3: Emotional abuse**				
**no (n=1009)**	8.99 (5.74)	2.61 (2.64)	26.97 (4.51)	13.09 (17.87)
**yes (n=77)**	12.19 (8.74)	3.84 (3.20)	27.37 (4.15)	11.60 (16.81)
**p value**	**0.0014****	**0.00025*****	0.429	0.081^{+}
**CTS item 4: Sexual abuse**				
**no (n=1026)**	9.05 (5.68)	2.64 (2.64)	27.01 (4.47)	13.18 (17.99)
**yes (n=60)**	11.98 (10.24)	3.68 (3.52)	26.86 (4.77)	9.72 (13.62)
**p value**	0.108	**0.036***	0.491	0.191
**CTS item 5 (inverse coding): Physical neglect**				
**no (n=883)**	8.80 (5.80)	2.57 (2.58)	26.76 (4.47)	12.60 (16.83)

**yes (n=203)**	11.04 (6.75)	3.27 (3.11)	28.04 (4.43)	14.68 (21.44)
**p value**	**0.000002*****	**0.008****	**0.000053*****	0.725

Notes: BMI: body mass index; CES-D: Center for Epidemiological Studies Depression Scale (Radloff, 1977; Hautzinger and Bailer, 1993); CI: confidence interval; CTS: Childhood Trauma Screener (Grabe *et al.*, 2012); GAD-7: Generalized Anxiety Disorder 7-item Scale (Spitzer *et al.*, 2006; Löwe *et al.*, 2008); N/n: sample sizes; SD: standard deviation.

$^{+}$ p ≤ 0.10; * p ≤ 0.05; ** p ≤ 0.01; *** p ≤ 0.001. Significant findings were marked in bold.

p values were based on Mann-Whitney U tests.

Table S2: Association of CTS scores reflecting different childhood trauma types and clinically relevant variables in participants of the study (N=1086)

Variables	Regression coefficient β (95% CI)	Stan-dardized ß	t	p value
**CES-D sum scores**				
**CTS item 1 (inverse coding): Severe emotional neglect (ref.: none)**	0.392 (-1.088; 1.871)	0.017	0.519	0.604
**CTS item 2: Medium/severe physical abuse (ref.: none)**	0.332 (-1.145; 1.808)	0.015	0.441	0.659
**CTS item 3: Medium/severe emotional abuse (ref.: none)**	2.070 (0.415; 3.725)	0.088	2.454	**0.014***
**CTS item 4: Sexual abuse (ref.: none)**	2.252 (0.672; 3.832)	0.085	2.796	**0.005****
**CTS item 5 (inverse coding): Severe physical**	1.915 (0.984; 2.846)	0.123	4.036	**0.00006*****

**neglect (ref.: none)**				
**GAD-7 sum scores**				
**CTS item 1 (inverse coding): Severe emotional neglect (ref.: none)**	0.105 (-0.560; 0.771)	0.010	0.310	0.757
**CTS item 2: Medium/severe physical abuse (ref.: none)**	0.237 (-0.427; 0.901)	0.024	0.700	0.484
**CTS item 3: Medium/severe emotional abuse (ref.: none)**	0.808 (0.064; 1.553)	0.077	2.130	**0.033***
**CTS item 4: Sexual abuse (ref.: none)**	0.781 (0.071; 1.492)	0.066	2.157	**0.031***
**CTS item 5 (inverse coding): Severe physical neglect (ref.: none)**	0.574 (0.155; 0.993)	0.083	2.690	**0.007****

Table S3: Co-occurrence of individual adversity subtypes in the total sample (N=1086)

Number	Severe emotional neglect	Physical abuse	Emotional abuse	Sexual abuse	Physical neglect	n	%
1	0	0	0	0	0	741	68.23
2	0	0	0	0	1	141	12.98
3	0	0	0	1	0	33	3.04
4	0	0	0	1	1	4	0.37
5	0	0	1	0	0	16	1.47
6	0	0	1	0	1	4	0.37
7	0	0	1	1	0	3	0.28
8	0	0	1	1	1	2	0.18
9	0	1	0	0	0	33	3.04
10	0	1	0	0	1	6	0.55
11	0	1	0	1	0	1	0.09
12	0	1	0	1	1	1	0.09
13	0	1	1	0	0	10	0.92
14	0	1	1	0	1	4	0.37
15	0	1	1	1	0	4	0.37
16	0	1	1	1	1	2	0.18
17	1	0	0	0	0	21	1.93
18	1	0	0	0	1	18	1.66
19	1	0	0	1	0	3	0.28
20	1	0	0	1	1	1	0.09
21	1	0	1	0	0	4	0.37
22	1	0	1	0	1	3	0.28
23	1	0	1	1	0	0	0

24	1	0	1	1	1	1	0.09
25	1	1	0	0	0	4	0.37
26	1	1	0	0	1	2	0.18
27	1	1	0	1	0	0	0
28	1	1	0	1	1	0	0
29	1	1	1	0	0	9	0.83
30	1	1	1	0	1	10	0.92
31	1	1	1	1	0	1	0.09
32	1	1	1	1	1	4	0.37

Notes: 0 = absent; 1 = present; n: group size.
